# Adiponectin Participates in Preeclampsia by Regulating the Biological Function of Placental Trophoblasts through P38 MAPK-STAT5 Pathway

**Published:** 2018-12

**Authors:** Gaoxia DONG, Ying TIAN, Xinqin LI

**Affiliations:** 1. Dept. of Obstetrics, The Second People’s Hospital of Liaocheng, Liaocheng 252600, China; 2. Dept. of Obstetrics, Zhangqiu Maternity and Child Care Hospital, Jinan 250000, China; 3. Dept. of Obstetrics, Jining First People’s Hospital, Jining 272011, China

**Keywords:** Adiponectin, MAPK, STAT5, Preeclampsia

## Abstract

**Background:**

We aimed to investigate the participation of adiponectin in preeclampsia, and to explore the possible mechanism.

**Methods:**

A total of 52 patients with preeclampsia and 30 normal women with full-term pregnancy were enrolled. Immunohistochemistry was used to detect the localization of MAPK and STAT5. RT-PCR was used to detect the expression of adiponectin mRNA in placental tissue of patients with preeclampsia and normal pregnant women. Western blot was used to detect the expression of adiponectin protein, MAPK, p-MAPK, STAT5 and p-STAT5 in placental tissue of patients with preeclampsia and normal pregnant women.

**Results:**

p–p38 was highly expressed in placental trophoblasts of patients with preeclampsia, while p-STAT5 was less expressed. Expression level of p–p38 and p-STAT5 in patients with preeclampsia were significantly different from those in normal pregnant women (*P*<0.01). Expression level of adiponectin mRNA was significantly lower in patients with preeclampsia than in normal pregnant women (*P*<0.05). Level of p–p38 expression was negatively correlated with levels of adiponectin expression (r=−0.413, *P*<0.05). Expression level of p-STAT5 was positively correlated with expression level of adiponectin (r=0.526, *P*<0.01).

**Conclusion:**

Adiponectin participates in preeclampsia by regulating the biological function of placental tropho-blasts through p38 MAPK-STAT5 pathway.

## Introduction

The incidence of preeclampsia is relatively higher (10%) (1). Preeclampsia is an idiopathic disease that causes higher mortality rate of pregnant women (2). At present, the pathogenesis of preeclampsia is still unclear, and the high incidence rate has attracted more and more attentions. Placental local functional abnormalities and placental trophoblastic function disorders may be responsible for pathophysiological changes of preeclampsia (3,4).

The predominant cause of preeclampsia is the presence of placenta, and more precisely to the presence of trophoblasts (3–5). Inadequate invasion of trophoblastic cells leads to an abnormal remodeling of spiral arteries, leading to the development of preeclampsia. Adiponectin is a cytokine that is specifically expressed in adipose tissue. Adiponectin is present in the serum mainly in the form of three different oligomers and is maintained at a high level, while serum adiponectin levels in patients with obesity, insulin resistance, type 2 diabetes mellitus, and atherosclerosis were significantly lower than those in normal subjects. Therefore, adiponectin was considered to be a protective factor for insulin resistance and vascular disease, and become a target for studies on preeclampsia (6, 7). Abnormal fluctuations of serum level of adiponectin were observed in patients with preeclampsia (8).

Placenta is an important organ that accompanies pregnancy. Its main components are the villi and trophoblasts that make up the villi. Because preeclampsia has the characteristic of remission or even disappearance of clinical manifestations after termination of pregnancy, it is suggested that the function of placental trophoblast cells may play a very important role in the pathogenesis of preeclampsia (9).

In this study, the expression of adiponectin in placental tissue of patients with preeclampsia and the expression of MAPK and STAT5 signaling pathways were explored, so as to lay a theoretical foundation for studies on the pathophysiology of preeclampsia.

## Materials and Methods

### General information

We included 52 patients from The Second People’s Hospital of Liaocheng, Liaocheng, China with preeclampsia diagnosed according to the diagnostic criteria for preeclampsia in obstetrics and 30 normal women with full-term pregnancy. Fifty two cases of preeclampsia were with a gestational age 34.3 ± 3.6 weeks, mean age of (25.4 ± 3.2) years, systolic blood pressure of 174.2 ± 7.5 mmHg and diastolic blood pressure of 113.5 ± 8.4 mmHg. Thirty cases of full-term pregnant women were with a gestational age of 39.5 ± 1.3 Weeks, mean age of (27.5±2.9) years, systolic blood pressure of 115.9±9.4 mmHg and diastolic blood pressure of 75.3±6.4 mmHg. Exclusion criteria: Presence of twin embryos and other adverse pregnancies, transfusion and immunotherapy, history of primary hypertension, hyperthyroidism, diabetes, and chronic nephritis (10).

The study was approved by the Ethics Committee of The Second People’s Hospital of Liaocheng and informed consents were signed by the patients.

### Experimental reagents

Rabbit anti-human p38, p–p38 MAPK, STAT5, p-STAT5 antibody were provided by Beijing HUAXIA YUANYANG Technology Co., Ltd. Goat anti-rabbit secondary antibody, and SP immunohistochemistry kit were from ShangHai Chaoyan Biotechnology Co., LTD. Enhanced chemiluminescence (ECL) for routine immunoblotting was from CLINX (Shanghai, China). DAB color development kit was purchased from Beijing Dingguo Changsheng Biotechnology Co., Ltd. Hematoxylin was frrom Beijing Sheng Dong Technology Co., Ltd. RNA extraction kit was from Beijing Tianmo Sci&Tech Development Co.,Ltd. Tissue protein extraction kit was from Shanghai Jingke Science and Technology Co., Ltd. BCA protein quantitation kit was from Nanjing SenBeiJia Biological Technology Co., Ltd. Reverse transcription kit was from Beijing Zhongtian Jingwei Technology Co., Ltd.

### Collection of placenta samples

Placenta tissue was collected form 52 patients with preeclampsia and 30 normal women with full-term pregnancy during cesarean section. Tissue (2cm*2cm*2cm) was collected from the root of umbilical cord. After washing with pre-cooled saline several times, tissue was fixed in 10% formalin solution. Then tissue was embedded with wax and stored at 4 °C.

### Detection of the localization of MAPK and STAT5 by immunohistochemistry

Paraffin-embedded tissue was cut to prepare 4 mm tissue sections. After dewaxing and hydration, tissue sections were treated with precooled PBS. Blocking was performed using 10% fetal bovine serum at room temperature for 15 min. Then tissue sections were then incubated with rabbit anti-human p–p38 MAPK and p-STAT5 antibodies overnight at 4 °C. After washing with precooled PBS several times, the corresponding goat anti-rabbit secondary antibody was added and incubated at room temperature for 30 min. After washing with precooled PBS several times, streptomycin - peroxidase was added and incubated at room temperature for 30 min. After washing with precooled PBS several times, DAB color treatment was performed. Then tissue sections were rinsed and sealed. Sections were observed under a microscope and IPP6.0 software was used to calculate the ratio of the positive area.

### QRT-PCR to detect the expression of adiponectin

Total RNA was extracted from fresh placental tissue according to the instructions of RNA extraction kit, and the concentration and purity of RNA samples were tested by spectrophotometer. Only RNA samples with an A260/A280 ratio of 1.9 ± 0.1 were used in reverse transcription. Primers of adiponectin and β-actin were synthesized by Genecreate (Wuhan, China). Reverse transcription reaction system was 20 μL (Table 1). PCR reaction system was 25μl and reaction conditions were: 94.0 °C for 3 min, followed by 40 cycles of 94.0 °C for 30 s, 61.0 °C for 3 s and 72.0 °C for 60 s, and 72 °C for 5 min. With β-actin as endogenous control, the relative expression level of adiponectin mRNA was calculated automatically by qRT-PCR machine.

**Table 1:** Primer sequences used in the study

***Adiponectin***	***Forward primer***	***5′-TGGCTGGAGTTCAGTGGTGTGA-3′***
β-actin	Reverse primer	5′-AACCAACCTGACGAATGTGGTGA-3′
	Forward primer	5′-TCACCCACACTGTGCCCATCTACGA-3′
	Reverse primer	5′-CAGCGGAACCGCTCATTGCCAATGG-3′

### Western blot to detect the expression of adiponectin protein

Total protein was extracted from fresh placental tissue according to the instructions of Protein Extraction Kit. Protein samples were stored at −70 °C before use. Protein samples were then subjected to electrophoresis using 8 or 10 % SDS-PAGE gel. Protein marker was used to identify the target protein. After transmembrane, membranes were blocked with 5% BSA solution for 90 min. Membranes were then incubated with p38, p–p38 MAPK, STAT5 and p-STAT5 primary antibodies (1:500) overnight at 4 °C. Then membranes were washed with precooled TBST solution three times, 5 min for each time, followed by incubation with corresponding goat anti-rabbit secondary antibody (1:1000) at room temperature for 1h. Then membranes were washed with precooled TBST solution three times, 5 min for each time. ECL solution was added and incubated in dark for 2 min. The results were scanned with ChemiDocTMMP imaging system, and Image J software was used to calculate the gray values.

### Statistical analysis

All statistical analyses were performed using SPSS17.0 software (Beijing Xinmeijiahong Technology Co., Ltd). Data were expressed as mean ± standard deviation. Comparisons between two groups were performed by *t* test. Pearson correlation analysis was used to test the correlation between the factors. α=0.05 was used as the test standard.

## Results

### Localization of MAPK and STAT5 expression in placental trophoblasts

**Fig. 1:**
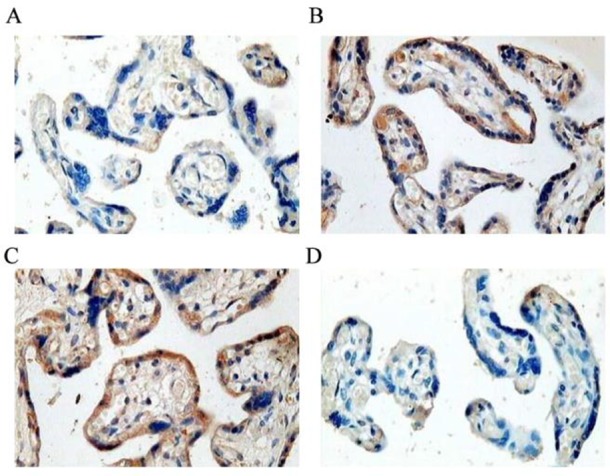
Localization of MAPK and STAT5 expression in placental trophoblasts of patients with preeclampsia (n=52) and normal pregnant women(n=30) (X40) A p–p38 expression in placental trophoblasts of normal pregnant women; B p–p38 expression in placental trophoblasts of patients with preeclampsia; C p-STAT5 expression in placental trophoblasts of normal pregnant women; D p-STAT5 expression in placental trophoblasts of patients with preeclampsia

**Fig. 2:**
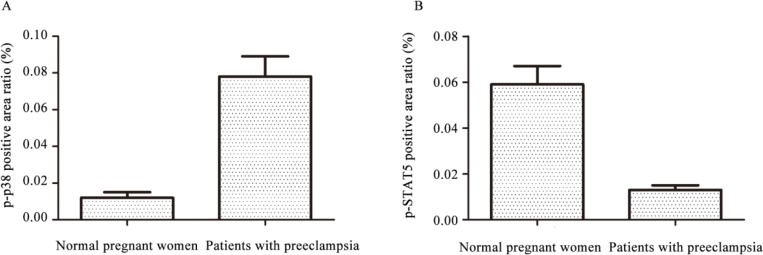
Relative expression levels of p–p38 and p-STAT5 in placental trophoblasts of patients with preeclampsia (n=52) and normal pregnant women(n=30) Notes: ^*^compared with normal pregnant women, *P*<0.05

Positive expression (brown granules) of p–p38 in placental syncytiotrophoblast of 52 patients with preeclampsia was strong and almost no staining of villous stromal was observed, while the expression in placental trophoblasts of 30 normal pregnant women was significantly weaker (0.012 ± 0.003 vs 0.078 ± 0.011, *P*<0.01). Positive expression of p-STAT5 in placental trophoblasts of patients with preeclampsia was weak, while the expression in placental trophoblasts of normal pregnant women was significantly stronger (0.059 ± 0.008 vs 0.013 ± 0.002, *P*<0.01) (Figs. 1 and 2).

### The expression of adiponectin mRNA and protein

**Fig. 3:**
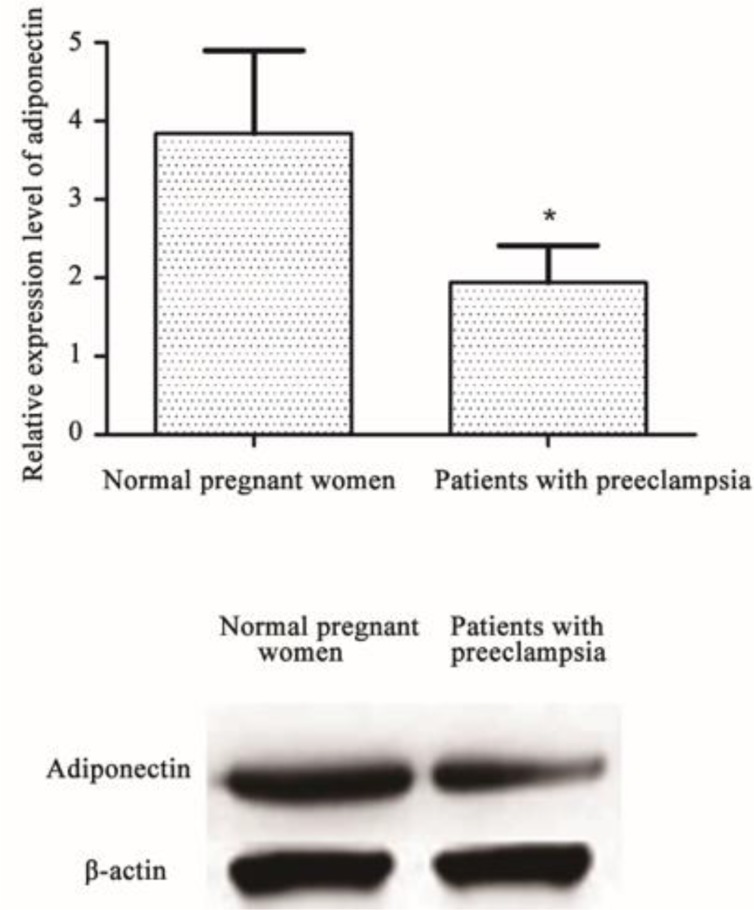
Relative mRNA and protein expression level of adiponectin in placenta of patients with preeclampsia (n=52) and normal pregnant women(n=30) Notes: ^*^compared with normal pregnant women, *P*<0.05

Expression of adiponectin mRNA was found in the placenta of both patients with preeclampsia and normal pregnant women, but there was a significant difference in expression of adiponectin mRNA and protein between the two groups. Expression level of adiponectin mRNA and protein was significant lower in patients with preeclampsia than in normal pregnant women (*P*<0.05) (Fig. 3).

### Expression of related proteins by Western blot

**Fig. 4:**
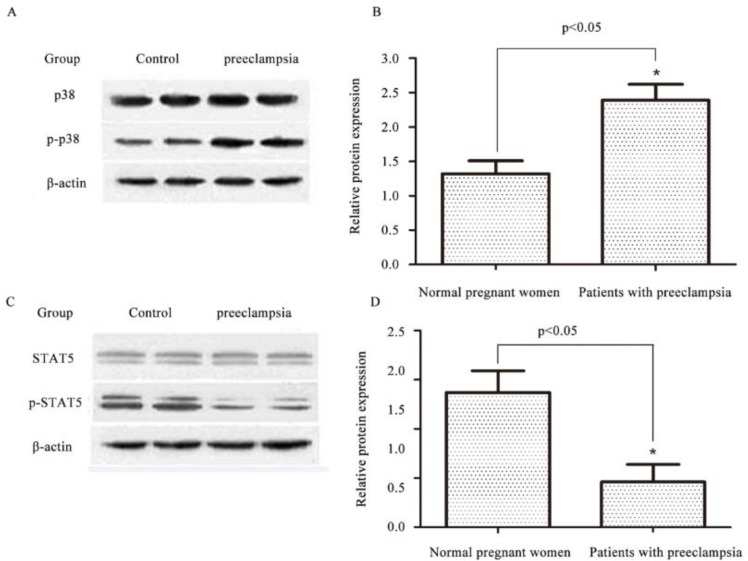
Expression of related proteins in patients with preeclampsia (n=52) and normal pregnant women(n=30) Notes: ^*^compared with normal pregnant women, *P*<0.05

No significant differences in expression levels of p38 and STAT5 were found between patients with 52 preeclampsia and 30 normal pregnant women. Expression level of p–p38 was significant lower in normal pregnant women than in patients with preeclampsia (*P*<0.01), while expression level of p-STAT5 was significant higher in normal pregnant women than in patients with preeclampsia (*P*<0.01) (Fig. 4).

### Correlation between the expression of adiponectin and the levels of p–p38 and p-STAT5

Correlations between adiponectin expression and p–p38 and p-STAT5 expression were tested by Pearson correlation analysis. The expression of adiponectin was decreased in preeclampsia, and expression of p–p38 was increased, which was negatively correlated with adiponectin expression (r=−0.413, *P*<0.05), while the expression level of p-STAT5 was decreased, which was positively correlated with adiponectin expression (r=0.526, *P*<0.05).

## Discussion

Preeclampsia is a unique gestational disorder that occurs in pregnant women after 20 weeks of gestation (11). Clinical manifestations of this disease include hypertension (12), proteinuria (13), and edema (14). Preeclampsia is a disease originates from placenta, and infiltration ability of placental trophoblasts will significantly reduce in pregnant women with preeclampsia (15,16).

Adiponectin expression in placental tissue of patients with preeclampsia is negatively correlated with many factors, such as gestational age, body mass index and urinary protein content, suggesting that the decrease in adiponectin expression may be associated with the occurrence of preeclampsia (17). However, the expression level of adiponectin in placental tissue gradually increases with the prolongation of gestational age, and expression level of adiponectin in placental tissue was significantly lower in patients with preeclampsia than in normal pregnant women with the same gestational age and body mass index (18). Soluble vascular endothelial growth factor receptor-1 (sFlt-1) is an endogenous angio-genesis inhibitor of the placenta (19). After placenta was delivered, concentration of sFlt-1 in bloodstream of the mothers was drastically reduced, showing that sFlt-1 was mainly derived from the placenta during pregnancy. sFlt-1 can compete with PLGF in the circulation to block the biological effects of PLGF and inhibit the formation of placental blood vessels, which can cause placental ischemia and hypoxia, and eventually develop into fetal growth restriction and affect pregnancy (20). The increase of sEndoglin in the circulation can lead to damage of vascular endothelium, and the central link in the onset of preeclampsia is maternal systemic disseminated endothelial cell injury. Therefore, sEndoglin is associated with preeclampsia (21). sEndoglin binds to TGF-β1 in the circulation, which in turn blocks the binding of TGF-β1 to TGF-βRII on the surface of vascular endothelial cells, so as to stop the transmission of TGF-β1 signal to the cells, resulting in vascular formation disorder and increased vascular permeability (22). sEndoglin binds to TGF-β1 in circulation, blocking the dephosphorylation of dephosphorylation of NOS (Thr495) by TGF-β1, resulting in inhibition of NOS activation, reduction of NO production, and vasoconstriction (23).

Immunohistochemical results of this study showed that positive expression of p–p38 in placental trophoblasts of patients with preeclampsia was strong, while the expression in placental trophoblasts of normal pregnant women was significantly weaker. In contrast, positive expression of p-STAT5 in placental trophoblasts of patients with preeclampsia was weak, while the expression in placental trophoblasts of normal pregnant women was significantly stronger. QRT-PCR detected expression of adiponectin mRNA in the placenta of both patients with preeclampsia and normal pregnant women, but there was a significant difference in expression of adiponectin mRNA between the two groups, and the expression level of adiponectin in patients with preeclampsia was significant lower than that in normal pregnant women. Results of Western blot showed that, there were no significant differences in expression levels of p38 and STAT5 between patients with preeclampsia and normal pregnant women. In contrast, expression level of p–p38 in normal pregnant women was significantly lower than that in patients with preeclampsia (*P*<0.01), while expression level of p-STAT5 in normal pregnant women was significantly higher than that in patients with preeclampsia (*P*<0.01). Expression of adiponectin was decreased in preeclampsia, and expression of p–p38 was increased, while the expression level of p-STAT5 was decreased, suggesting that p–p38 expression was negatively correlated with adiponectin expression and p-STAT5 expression was positively correlated with adiponectin expression.

## Conclusion

The reduced expression level of adiponectin mRNA in patients with preeclampsia may participate in the development of preeclampsia by up-regulating p–p38 expression and downregulating p-STAT5 expression.

## Ethical considerations

Ethical issues (Including plagiarism, informed consent, misconduct, data fabrication and/or falsification, double publication and/or submission, redundancy, etc.) have been completely observed by the authors.
